# Strong Correlations between Social Appearance Anxiety, Use of Social Media, and Feelings of Loneliness in Adolescents and Young Adults

**DOI:** 10.3390/ijerph20054296

**Published:** 2023-02-28

**Authors:** Triada Konstantina Papapanou, Christina Darviri, Christina Kanaka-Gantenbein, Xanthi Tigani, Maria Michou, Dimitrios Vlachakis, George P. Chrousos, Flora Bacopoulou

**Affiliations:** 1 Postgraduate Course of Science of Stress and Health Promotion, Medical School, National and Kapodistrian University of Athens, 115 27 Athens, Greece; 2 University Research Institute of Maternal and Child Health & Precision Medicine, Medical School, National and Kapodistrian University of Athens, 115 27 Athens, Greece; 3 Center for Adolescent Medicine and UNESCO Chair in Adolescent Health Care, Aghia Sophia Children’s Hospital, Medical School, National and Kapodistrian University of Athens, 115 27 Athens, Greece; 4 First Department of Pediatrics, Aghia Sophia Children’s Hospital, Medical School, National and Kapodistrian University of Athens, 115 27 Athens, Greece; 5 Human Ecology Laboratory, Department of Home Economics and Ecology, Harokopio University, 176 71 Athens, Greece; 6 Laboratory of Genetics, Department of Biotechnology, School of Applied Biology and Biotechnology, Agricultural University of Athens, 118 55 Athens, Greece; 7 Lab of Molecular Endocrinology, Center of Clinical, Experimental Surgery and Translational Research, Biomedical Research Foundation of the Academy of Athens, 115 27 Athens, Greece

**Keywords:** social appearance anxiety, social media, internet, loneliness, adolescents, young adults

## Abstract

Social appearance anxiety is a type of social anxiety that is associated with body image perception and exacerbated by the use of social media, leading to feelings of loneliness. The purpose of this cross-sectional study was to examine the relations between social appearance anxiety, use of social media, and feelings of loneliness in Greek adolescents and young adults. The sample of the research consisted of 632 participants, 439 women (69.5%) and 193 men (30.5%), aged 18–35 years. The Social Appearance Anxiety Scale (SAAS), the Social Media Disorder Scale (SMDS), and the UCLA Loneliness Scale were the instruments used. Data collection was performed online, via Google forms. Multiple regression analyses were performed and demonstrated a significant positive correlation between the Social Appearance Anxiety Scale and the UCLA Loneliness Scale scores. The feeling of loneliness was predicted by the social appearance anxiety score (*p* < 0.0001). On the other hand, there was a significant negative correlation between the Social Appearance Anxiety Scale and the Social Media Disorder Scale scores (*p* = 0.002), suggesting that social media use may exacerbate appearance anxiety and, hence, loneliness. The findings suggest that there may be a complex, vicious reverberatory cycle between appearance anxiety, use of social media, and feelings of loneliness in some young people.

## 1. Introduction

The coping strategies that young people employ regarding their body image and social anxiety are among the main factors with a direct impact on both their identity development and their intimate relationships [1]. Social anxiety is specifically related to a lack of confidence in one’s presentation abilities and a desire to make a favorable impression on others [2]. Social appearance anxiety, on the other hand, is a type of anxiety defined as the fear of being negatively evaluated or rejected by others because of one’s physical appearance [3,4,5,6,7]. People who experience social appearance anxiety usually hold a negative perception of their body and appearance [8,9,10,11], and they frequently try to camouflage the features or the body parts they dislike [8]. In addition, they prefer minimum physical contact and instead use internet communication, which involves less risk of exposure, as well as offering an easier way to manipulate their image and overall appearance [2]. Anxiety about one’s social appearance can create a strong desire not to be seen [12], and a fear of rejection due to appearance can exacerbate feelings of loneliness [13]. Social media provides the opportunity for individuals to be seen in the way that they desire [12]. People who experience severe social appearance anxiety seek communication that does not expose them socially to avoid this anxiety. Therefore, they turn to the use of the internet, which does not expose them to such a great extent and enables them to cover those aspects of their appearance that they consider unattractive [2].

The internet attracts more users every day, and it has an impact on many aspects of people’s lives, frequently becoming an essential part of their daily activities [14]. Young women’s and men’s body image concerns and use of social media are two concepts that are directly intertwined [15]. The internet and social media contribute to many types of social comparison, creating an ideal body image. On the internet, people are exposed to a multitude of images that may be considered the most suitable as a benchmark for social appearance [8]. Many of them are idealized and associated with negative feelings about the body and the person’s desire to change their weight and body shape [16]. Social media gives messages about what the bodies of men and women should look like. For women the ideal body is lean, while for men it is muscular. People who are not happy with their appearance and use the internet experience more stress and symptoms of depression, as well as more problems with internet use [17]. People upload the best version of themselves to social media, the most attractive photos, which they may have first edited and improved. However, beyond photos, they share content that concerns their appearance and may receive comments—a fact that is likely to have an effect on how they feel about their appearance [15].

Social networking is the most popular choice for social interaction [12,18]. Through platforms such as Facebook, people expose themselves and their lives to others, who in turn respond with a negative or positive evaluation. On social media platforms, people are exposed to images of others and social comparison often occurs, which can easily exacerbate concerns about appearance [8,19]. These concerns are minimized when there is positive feedback and increased in the case of negative comments [19]. Many young people tend to internalize false body ideals that make them feel that they fall short and, hence, experience dissatisfaction with their own body and appearance [20,21]. Therefore, use of social media has been strongly associated with social anxiety [1]. Some studies have shown that people with higher levels of social anxiety use social media more often [22]. In particular, people with social appearance anxiety [12] and those who are more anxious about face-to-face contact are more regular users [23]. 

Loneliness is an emotional state observed in all age groups, even in adolescents and young adults, and varies with age [24]. Young people who perceive themselves as less physically attractive tend to express higher levels of loneliness [25], while increased levels of loneliness have an impact on the time they spend on screens and on social media [26,27]. The levels of loneliness are predicted by social appearance anxiety [28,29], and about 10% of the level of loneliness is explained by social appearance anxiety [30]. Loneliness is influenced by appearance rejection sensitivity [13].

Recent findings on the above issue are contradictory, as some studies support the notion that the feelings of loneliness are increased because of the use of social media [31], while others support the exact opposite finding [32]. Regardless of these findings, however, social appearance anxiety and loneliness are significant predictors of social media usage [12]. Another aspect yet to be further clarified is whether people who spend more time on social media are more concerned about their appearance, or whether people who are more concerned with their appearance spend more time on social media [15]. Regardless of these questions, an undeniable fact is that the internet and social media platforms are a major component of modern life [33] and, despite the profound benefits and opportunities they provide, their increasing use and misuse can lead to serious behavioral dependence and psychological problems [14,34]. 

Research has shown that both genders do not feel satisfied with their body from a young age and even into maturity, and some would like to proceed to change some parts of their body [35]. Body dissatisfaction is mainly observed in females, and covers the entire age range from adolescence to adulthood [36]. Regarding gender, there have been conflicting findings as to gender differences in social appearance anxiety, with some authors finding no differences [3,25] and others reporting that women experience more anxiety about their social appearance than men [19,37,38,39], whereas one study found that males experienced more anxiety about their social appearance than females [40].

It also seems to be of more concern to younger people who, on the one hand, wish to improve their body image and, on the other hand, share images of their bodies with their online friends [19]. Body image concerns appear from childhood and are particularly enhanced in adulthood. Social appearance is currently a topic of discussion among individuals of all ages, particularly young people. 

Therefore, the aim of this study was to investigate the relations between social appearance anxiety, the use of social media and feelings of loneliness. More specifically, it aimed to examine how social appearance anxiety is affected by the use of social media and loneliness, and to define the potential gender differences in these relations in a Greek sample of adolescents and young adults aged 18–35 years old.

## 2. Materials and Methods

### 2.1. Research Design

A cross-sectional design was employed to examine the relations between the “Social Appearance Anxiety Scale”, “the use of social media” and “the feeling of loneliness” in a sample of Greek young adults aged 18–35 years.

### 2.2. Participants, Procedures

The study used a convenience sample. Participants’ inclusion criteria were as follows: (1) using a social media platform, (2) being 18–35 years old and (3) being able to read and write in the Greek language. Individuals diagnosed with a severe mental disorder and taking medication were barred from participating.

### 2.3. Data Collection 

The data collection was conducted between the 2nd of March and the 30th of July, 2022. The survey was conducted via the internet and the anonymity of the participants was preserved. The instruments were distributed online through various social media platforms using Google forms in various groups. The estimated completion time for all 3 questionnaire scales was 10–15 min. Prior to research, investigators obtained written author permission to use all the different scales applied.

### 2.4. Ethics Approval

This study was approved by the Bioethics committee of the Medical School of the National and Kapodistrian University of Athens (approval protocol number 611). All participants were fully informed about the aims and the procedures of the study, and all provided their informed consent by ticking the corresponding box. Participants were free to withdraw from the study at any point.

### 2.5. Measures 

Sociodemographic and Anthropometric Characteristics Questionnaire: Variables include information about gender, age, height, weight, marital status, education level and occupation, as well as two questions regarding use of social media and the average time of use per day. In addition, height and weight were self-reported by participants. Body mass index (ΒΜΙ) was calculated using the following formula BMI = weight/height^2^.

Social Appearance Anxiety Scale (SAAS): This scale was originally developed by Hart et al. (2008) and examines the fear of being negatively judged by others based on one’s overall appearance. It consists of 16 items; answers were given on a 5-point Likert scale with a range from 0 (not at all) to 5 (extremely). The total score ranges from 18 to 80. Those who score high on the scale experience higher social appearance anxiety. The SAAS demonstrated good internal consistency in all three samples: α = 0.94, 0.95 and 0.94, respectively. In this study, the Greek language validated adaptation was used [41].

Social Media Disorder Scale (SMDS): This scale consists of a total of 9 items. Answers are provided in a binary form (yes/no) referring to the period of the last 12 months. This scale has been determined efficient in measuring social media addiction. The internal consistency ranged between 0.76 and 0.82 [42]. The Greek language validated adaptation was used [43].

UCLA Loneliness Scale: This scale measures subjective feelings of loneliness and social alienation/isolation. It consists of 20 items, 10 positive and 10 negative statements, which are rated on a 4-point Likert scale, ranging from 1 (never) to 4 (often). The internal consistency was a = 0.90 [44]. Higher scores indicate a higher level of loneliness and social isolation. In this study, the validated Greek language adaptation was employed [45].

### 2.6. Statistical Analysis

All statistical analyses were performed using IBM SPSS (version 24.0) for Windows. Descriptive analysis was conducted for categorical and continuous variables. The Mann–Whitney non-parametric test was used to evaluate differences in continuous variables between men and women, due to the skewed distribution of the continuous variables. For the comparison between categorical variables, Pearson chi square test was used. Multiple linear regression analysis was used to evaluate various participant characteristics and measures (independent variables) as determinants of SAAS (dependent variable). The results are presented as unstandardized b ± coefficients (standard error, *p*-value). Collinearity was tested using the variance inflation factor (VIF). All variables had a value of <4, suggesting no presence of collinearity.

## 3. Results

The study sample included 632 participants aged between 18 and 35 years (Mean 25.54, SD 4.54). Table 1 presents the sociodemographic characteristics of the sample. Most of the subjects were women (69.5%), unmarried or divorced (91.1%), with tertiary education (46%). Regarding occupational status, most of the participants were university students (34%) or private employees (30.9%). The mean BMI score was 23.27 kg/m^2^ (SD 4.20). With regards to gender differences, women reported significantly higher education levels (*p* = 0.007), higher marriage status (*p* = 0.021) and higher unemployment status (*p* = 0.010). Men, on the other hand, had significantly higher BMI scores (*p* < 0.0001) than women.

Table 2 presents the total scores of the instruments used (SAAS, SMDS, UCLA) and gender-based differences in scores. Statistically significant differences were observed in both the SAAS and SMDS questionnaires. More specifically, women scored higher in SAAS and lower in SMDS than men (*p* < 0.0001 for both instruments). 

Table 3 presents the correlations between study instruments. SAAS was negatively correlated with the SMDS score (r = −0.292, *p* < 0.0001) and positively correlated with the UCLA score (r = 0.392, *p* < 0.0001). Finally, UCLA was negatively correlated with SMDS (r = −0.225, *p* < 0.0001).

Table 4 presents the results of the linear regression analysis for social appearance anxiety in relation to various participant characteristics and measurements. Gender was significantly associated with levels of SAAS. Specifically, men reported lower levels of SAAS by −5.502 points (*p* < 0.0001) in comparison to women. Higher levels of BMI and UCLA were positively associated with higher levels of SAAS, by 0.439 points (*p* < 0.0001) and 0.511 points (*p* < 0.0001), respectively. Finally, levels of SMDS were negatively associated with levels of SAAS, with −0.768 points (*p* = 0.002).

## 4. Discussion

Our study investigated the relations between social appearance anxiety, the use of social media and feelings of loneliness in a Greek sample. In our analysis, social appearance anxiety was the dependent variable, while the use of social media, feelings of loneliness, and gender were independent variables. Overall, these findings support the notion that social appearance anxiety, use of social media and feelings of loneliness are all correlated with each other. Social appearance anxiety was predicted by the use of social media, feelings of loneliness, BMI and gender.

Regarding gender-based differences, the analysis showed that women had higher levels of social appearance anxiety than men. This difference has been observed by other researchers [37,38,39,46]. An earlier study showed that social appearance anxiety was significantly higher in women than men, and that there was a positive relation between social appearance anxiety and social anxiety in relationships, fear of negative evaluation and neuroticism [39]. Furthermore, in a study of a clinical population with acne vulgaris, females scored significantly higher in social appearance anxiety than males [25,47]. Nevertheless, other studies did not find any sex differences in the SAAS levels [3,25,48], while one study found that the levels of SAAS were higher in males than females [40].

Another significant finding of our study was the positive relation between social appearance anxiety and body mass index (BMI). The social appearance anxiety was predicted by BMI. This finding is also in line with previous studies that found a positive correlation between SAAS and BMI, with people with a high BMI having higher scores than those with a low BMI [40,49].

Our study also demonstrated a significant negative correlation between SAAS and SMDS. This means that an increase in SMDS was associated with a decrease in the SAAS. The use of social media was a significant predictor of social appearance anxiety. This finding is supported by a previous study, in which social media usage and social appearance anxiety were negatively correlated. This might be due to social media trends such as “body positivity”. Numerous social media influencers and public personalities address body image concerns and promote body positivity. Social media may also be used to engage with other users and to reach good and healthy material. Body positivity on social media might be one way to increase healthy body satisfaction and confidence. An important advantage of social media platforms is that they connect users to the achievement of specific goals, leading to a positive social transformation. Those who are not satisfied with the way they look in the mirror possibly to turn to the use of the internet [50]. When users receive positive feedback for their image on social media, their physical-appearance-based self-esteem level is increased, and so they do not experience social appearance anxiety [12].

According to previous studies, problematic internet use and social appearance satisfaction are correlated negatively [1,17,23]. On the other hand, many studies have found a significant positive correlation between social appearance anxiety and internet addiction. Social appearance anxiety acts as a predictor of internet addiction [14,16]. Other studies have shown that social appearance anxiety, internet addiction and social addiction are positively correlated in young adults [8,51]. Doğan and Çolak (2016) [12] have shown that the use of social media is positively correlated with social appearance anxiety and is a predictor of social network usage.

The exposure to the thin, ideal body image or idealized body pictures is possibly increasing body dissatisfaction and causing stress, embarrassment and depressive feelings. This may influence the onset of social appearance anxiety [52]. Individuals who are exposed to visual information in social media may create more social comparisons. Online activities focused on body image may contribute to problematic use of social media because people who are disappointed with their appearance develop and control the way they present themselves on the internet [19]. According to Aslan and Tolan (2022), there is a positive relationship between social appearance anxiety and social media addiction. Social appearance anxiety is a predictor of social media addiction. Furthermore, there is a considerable association between media usage and social appearance anxiety in women [10].

In line with the literature [53], we found a significant positive correlation between social appearance anxiety and feelings of loneliness. Feelings of loneliness were a significant predictor of social appearance anxiety, meaning that as feelings of loneliness increase, so does the anxiety experienced by individuals regarding their social appearance. Thus, as the level of SAAS increases, so does the level of loneliness. Social appearance anxiety may create difficulties with socialization, and may cause a preference for being alone [25]. There are some factors, such as negative thoughts about themselves and others, experiences of social rejection and social anxiety, that minimize the possibilities of developing meaningful social relationships with people who are socially anxious and lonely. This causes them to be more vulnerable to social isolation and puts them at an increased risk of future loneliness [27,54,55,56]. Concerns about physical appearance cause anxiety about the possibility of rejection due to decreased physical attractiveness. Fear of rejection due to appearance exacerbates feelings of loneliness [12,13].

We found a significant negative relation between the use of social media and loneliness, and this is consistent with the research by Shaw and Gant (2002) [32]. Similarly, Singh et al. agreed that there is a negative correlation between internet addiction and loneliness [29]. On the other hand, use of the internet can reduce the feelings of loneliness among users as a result of personal trait variables, and can increase measures of social support [32]. In contrast, there are studies that support the opposite finding. Specifically, it was found that the use of social network sites increased the levels of loneliness [31]. In addition, loneliness was a predictor for internet addiction. Individuals who were addicted to the internet were socially anxious and emotionally lonely [57]. The loneliness was associated with increased levels of internet use [58], but use of the internet decreased the levels of loneliness when there was positive communication [59].

Loneliness could result in addiction to social media [60], but the opposite finding was also observed [14]. Some studies suggested a direct influence of loneliness on the use of social networking sites [12,61,62,63]. Individuals with feelings of loneliness may be driven to use the internet due to their desire for companionship. The anonymity and lack of face-to-face communication that is afforded by social media may lead to a decrease in social anxiety [58]. The anxiety that someone experiences for their social appearance can create a stronger desire to not be seen. Social media provides people with the ability to be viewed as they wish [12]. A positive correlation between social media addiction, social anxiety and loneliness was described, but the social media addiction and loneliness correlation was weak. Social anxiety was a predictor of social media addiction, but loneliness was not a predictor for social media addiction [14]. We found that social appearance anxiety was a predictor of loneliness, but not of usage of social media.

This study has some limitations. The sample was limited to the age group of adolescents and young adults, so the results cannot be generalized to other ages. Further surveys could include adults of all ages. The time that people spent on social networks was not included in the analysis, as this information was provided by few participants. In addition, the sample was unbalanced in terms of gender, with the number of women participating being higher than that of men. Another limitation is that this was a cross-sectional study. Longitudinal studies should be done, and different factors should be added.

## 5. Conclusions

This study demonstrated the association of social media use, social appearance anxiety and feelings of loneliness. We found that both the use of social media and feelings of loneliness were predictive of social appearance anxiety. There could be a vicious circle connecting social appearance anxiety, social media use and loneliness among certain young individuals. To investigate the causal mechanisms behind these connections, additional studies are required.

## Figures and Tables

**Table 1:** Sociodemographic characteristics (N = 632).

	Men	Women	*p*-Value	Total
Gender N (%)	193 (30.5)	439 (69.5)	-	632 (100)
Age Mean (SD)	25.42 (4.45)	25.59 (4.58)	0.905	25.54 (4.54)
Median (IQR)	25 (7)	25 (7)		25 (7)
**Education level N (%)**			0.007	
- Upper Secondary Education (High School)	77 (39.9)	124 (28.2)		201 (31.8)
- Tertiary Education	84 (43.5)	207 (47.2)		291 (46.0)
- Advanced Tertiary Education (MSc/PhD)	32 (16.6)	108 (24.6)		140 (2.2)
**Marital Status N (%)**			0.021	
- Unmarried/Divorced	184 (95.3)	392 (89.3)		576 (91.1)
- Married	9 (4.7)	47 (10.7)		56 (8.9)
**Job Status N (%)**			0.010	
- Public Employee	22 (11.4)	56 (12.8)		78 (12.3)
- Private Employee	71 (36.8)	124 (28.2)		195 (30.9)
- Freelancer	25 (13)	42 (9.6)		67 (10.6)
- University Student	57 (29.5)	158 (36)		215 (34)
- Unemployed	10 (5.2)	51 (11.6)		61 (9.7)
- Other	8 (4.1)	8 (1.8)		16 (2.5)
**Body Mass Index (BMI)**			<0.0001	
Mean (SD)	25.32 (3.93)	22.37 (4.00)		23.27 (4.20)
Median (IQR)	25.06 (4.40)	21.45 (4.16)		22.49 (5.12)

*p* < 0.05, ×2.

**Table 2:** Scores of instruments used and differences according to participants’ gender (N = 632).

	Men	Women	*p*-Value	Total
**SAAS Total**			<0.0001	
Mean (SD)	31.28 (13.55)	36.12 (13.37)		34.64 (13.60)
Median (IQR)	27 (17)	34 (18)		32 (19)
**SMDS Total**			<0.0001	
Mean (SD)	16.05 (2.16)	15.58 (1.93)		15.72 (2.01)
Median (IQR)	17 (3)	16 (3)		16 (2)
**UCLA Total**			0.809	
Mean (SD)	37.40 (9.82)	37.25 (10.01)		37.30 (9.95)
Median (IQR)	36 (17)	35 (15)		36 (15.75)

*p* < 0.05, Mann–Whitney.

**Table 3:** Correlations between measures.

Spearman’s Rho	SAAS	SMDS	UCLA
SAAS	**1**		
SMDS	−0.292 *	**1**	
UCLA	0.392 *	−0.225 *	**1**

* Correlation is significant at the 0.01 level (2-tailed).

**Table 4:** Results (b, SE) from regression analysis models that evaluated various participant characteristics and measures as determinants of SAAS.

	b ± SE, *p*	Statistics VIF
Gender (Man/Woman)	−5.502 ± 1.130, <0.0001	1211
Age	−0.229 ± 0.166, 0.169	2543
Marital Status (Married/Unmarried/Divorced)	−1.672 ± 1.901, 0.379	1303
Education Level (Ref: Secondary Education)		
- Tertiary Education	−0.370 ± 1.355, 0.785	2037
- Advanced Tertiary Education (MSc/PhD)	−0.164 ± 1.797, 0.927	2489
Job Status (Ref: Public Employee)		
- Private Employee	−0.350 ± 1.639, 0.831	2559
- Freelancer	−3.393 ± 2.007, 0.091	1705
- University Student	0.912 ± 1.927, 0.636	3721
- Unemployed	3.647 ± 2.113, 0.085	1740
- Other	−3.431 ± 3.356, 0.307	1241
BMI	0.439 ± 0.121, <0.0001	1158
SMDS	−0.768 ± 0.247, 0.002	1101
UCLA	0.511 ± 0.050, <0.0001	1084

## Data Availability

According to the ethics approval, data can only be accessed by the members of the research team.
